# Cervical Infection with *Herpes simplex* Virus, *Chlamydia trachomatis*, and *Neisseria gonorrhoeae* among Symptomatic Women, Dubai, UAE: A Molecular Approach

**DOI:** 10.1155/2014/347602

**Published:** 2014-05-27

**Authors:** Davood Mehrabani, Mohammad Amin Behzadi, Saeed Azizi, Hamid Payombarnia, Ali Vahdani, Mandana Namayandeh, Mazyar Ziyaeyan

**Affiliations:** ^1^Stem Cell and Transgenic Technology Research Center, Shiraz University of Medical Sciences, Shiraz 71345-1744, Iran; ^2^Iranian Hospital, Dubai 2330, UAE; ^3^Professor Alborzi Clinical Microbiology Research Center, Namazi Hospital, Shiraz University of Medical Sciences, Shiraz 7193711351, Iran

## Abstract

Tragically, genital tract infections are still a major public health problem in many regions. This study was undertaken to determine the prevalence of cervical infection with *Herpes simplex* virus (HSV), *Chlamydia trachomatis* (CT), and *Neisseria gonorrhoeae* (NG) among married women referring to Iranian Hospital, Dubai, UAE. In a retrospective cross-sectional survey, 201 female patients aged 16–80 years who referred to the Obstetrics and Gynecology Department of Iranian Hospital, Dubai, UAE, in 2010 were enrolled. The patients were categorized into three age groups: 15–30 (group I), 31–40 (group II), and ≥41 years old (group III). A cervical swab sample was collected from each woman and the prevalence of cervical infection with HSV, CT, and NG was determined by PCR method. HSV, CT, and NG were detected in 6.5%, 10.4%, and 5.5% of swab samples, respectively. Regarding age, a significant difference was noticed for prevalence of NG and HSV between groups I and III. Because of public health importance of sexual transmitted diseases (STDs), their long-lasting impact on quality of life, and their economic burden, preventing measures and education of women seem necessary.

## 1. Introduction

Tragically, genital tract infections are still a major public health problem in many regions [1]. Annually in the United States, more than 15 million new cases of sexually transmitted diseases (STDs) were reported [2]. Among females, some of them may lead to long-term pelvic inflammatory diseases, infertility, ectopic pregnancies, dyspareunia, and cervical cancer [3, 4]. Every year, more than half of all new cases of STDs occur in young individuals between 15 and 24 years [5]. In addition, in young and adolescent women, they may result in depression, low social support, and prominent stress [6].

The majority of STDs such as* Herpes simplex* virus (HSV),* Chlamydia trachomatis* (CT), and* Neisseria gonorrhoeae* (NG) were shown to be asymptomatic in women [4, 7, 8]. Genital Herpes is generally considered as a common chronic STD in both developed and developing countries with substantial morbidity [9, 10]. The infection is caused by two types of virus including HSV-2 (mainly) and HSV-1 (sometimes) [11] while their prevalence and incidence have increased in the past three decades [11].

CT infection is the most frequent cause of bacterial STD in the world, especially in young women [12]. The infection is asymptomatic in most cases and can be transmitted during vaginal, oral, or anal sexual contact and can be passed by the mother to newborn too [4].

Gonorrhea is caused by NG and this pathogen was demonstrated to have the potential to develop resistance to frequently used antimicrobial agents, especially in uncured patients. These cases may continue to transmit and facilitate the rapid emergence of antimicrobial resistance [13]. The incidence rate of the disease is high; therefore diagnosis of both symptomatic and asymptomatic infections is of great importance [14].

In recent years, rapid detection kits and dipsticks have been used as common, convenient, and fast methods of screening for STDs. However, new molecular methods such as PCR, qualitative and quantitative real time PCR, and DNA hybridization were introduced as more reliable techniques in diagnosis of a wide variety of STDs in comparison to serological methods. Moreover, monitoring of DNA level of a pathogen in body fluids can reveal the status of the disease, its response to medication, and its resistance patterns [15].

In Dubai, UAE, the individuals' lifestyle has changed. In addition, many migrant workers or investors were attracted from more than 100 countries from Asia, Africa, Europe, and so forth to this city. As Dubai is the 8th most visited city in the world by tourists and was shown to attract more than 15 million tourists from various countries till 2015, therefore, STDs can be an emerging public health concern in Dubai, UAE. Screening of STDs in Dubai population can be the first and very critical step of managing a public health problem. The objective of this study was to determine the prevalence of cervical infection with HSV, CT, and NG among married women referring to Iranian Hospital, Dubai, UAE.

## 2. Subjects and Methods

### 2.1. Study Population

The study involved 201 female patients aged 16–80 years old (33.21 ± 9.71 years) who referred to Obstetrics and Gynecology Department of Iranian Hospital, Dubai, in 2010 with symptoms such as itching in genital area, dyspareunia, dysuria, or abnormal vaginal discharges. The patients were from different nationalities (Table 1) and all of them were married. The patients were categorized into three age groups: 15–30 (group I), 31–40 (group II), and ≥41 years old (group III). A cervical swab sample was collected by sterile swabs from all patients and was transferred in a viral transport medium to the Professor Alborzi Clinical Microbiology Research Center, Nemazee Hospital, Shiraz, Iran, for further investigation. The study was approved in Office of Education and Research of Iranian Hospital, Dubai.

### 2.2. DNA Extraction

DNA was extracted from swab samples in 200 *μ*L of viral transport medium by Invisorb spin virus DNA Mini Kit (Invitek, Berlin, Germany) according to the manufacturer's protocol. For detection of CT, a standardized amount of internal control DNA, supplied with the real time PCR kit, was added to the lysis buffer kit to monitor the efficiency of extractions. Negative and positive controls were included in the extraction process.

### 2.3. Real Time Quantitative PCR for Detection of HSV and CT

The real time quantitative PCR was performed using oligonucleotide primer pairs and probes specific for the region of HSV1 and HSV2 glycoprotein B (gB), as reported previously [15]. The primers used were HSVFP (5′TCC CGG TAC GAA GAC CAG3′) and HSVRP (5′AGC AGG CCG CTG TCC TTG3′), and the probe was HSVTCP (5′FAMTGG TCC TCC AGC ATG GTG ATG TTG/C AGG TCGTAMRA3′). The reaction was carried out with the following protocol: 2 min of incubation at 50°C for AmpErase activation, 10 min at 95°C for polymerase activation and for 45 cycles, 15 seconds at 94°C for denaturation, and 60 seconds at 58°C for annealing, extension, and data collection. Each 50 *μ*L of PCR mixture contained 10 *μ*L of purified DNA, 840 nM concentration of each primer, and 100 nM probe in 1x TaqMan universal PCR master mix (Applied Biosystems, Branchburg, New Jersey, USA).

For detection of CT, a real time quantitative PCR was carried out with oligonucleotide primer pairs and probe specific for CT genome by Advanced Kit (PrimerDesign Ltd., Millbrook Technology Campus, South Hampton, UK). Amplification was performed using TaqMan universal real time PCR master mix reagents (Roche, Branchburg, New Jersey, USA). It was done with the following four steps protocol: 2 min of incubation at 50°C for AmpErase activation, 10 min at 95°C for polymerase activation and for 45 cycles, 10 seconds at 95°C for denaturation, and 60 seconds at 60°C for annealing, extension, and data collection.

All amplifications were carried out in an Applied Biosystem Sequence Detector 7500 machine (Applied Biosystems, USA). Negative controls were included in the extraction process between every 20 clinical samples. All of the negative samples were tested twice.

### 2.4. Detection of NG

NG was detected with a PCR detection kit (CinnaGen Inc., Iran) according to the manufacturer's protocol. The reaction mixture was heated at 94°C for 3 min and then incubated for 35 cycles of 94°C for 45 seconds, 50°C for 20 seconds, and 72°C for 30 seconds and for 5 min at 72°C for an additional extension. The PCR products were analyzed on 1% agarose gel.

### 2.5. Statistical Analysis

Differences in prevalence of HSV, CT, and NG between age groups were analyzed with chi-square test. Moreover, the association between HSV, CT, and NG and the occurrence of fungal-bacterial cervical infection were analyzed with chi-square test. The entire data were analyzed by SPSS software (SPSS for Windows, version 16, SPSS Inc., Chicago, IL, USA). All values of *P* < 0.05 were considered statistically significant.

## 3. Results

*Candida* spp. and* Coccobacilli* cervical infection were considered as fungal-bacterial infection with a prevalence of 45.3% and non-fungal-bacterial infection with a prevalence of 54.7%. HSV and CT were positive in 13 (6.5%) and 21 (10.4%) swab samples, respectively. The copy number of HSV DNA measured by the real time PCR assay ranged from 3.11 × 10^3^ to 7.18 × 10^5^ copies/mL in the viral transport medium (median of 4 × 10^4^ copies/mL) and CT DNA copy number ranged from 3.48 × 10^3^ to 7.11 × 10^6^ copies/mL in the viral transport medium (median of 3.33 × 10^5^ copies/mL). NG was detected in 11 (5.5%) patients.

Table 2 presents the prevalence of the diseases in different age groups. None of the patients had mixed infections. There were no significant differences in prevalence of CT between different age groups (*P* > 0.05). The prevalence of NG and HSV was not significantly different between groups I and II and II and III (*P* > 0.05); however, there were significant differences between groups I and III (*P* < 0.05). Moreover, the differences in prevalence of HSV, CT, and NG infections were not statistically significant between fungal-bacterial cervical infection group and non-fungal-bacterial group (*P* > 0.05).  Table 3 compares our data in Dubai, UAE, for prevalence of HSV, CT, and NG with different studies.

## 4. Discussion

In the current study, out of 201 women, 6.5%, 10.4%, and 5.5% were infected with HSV, CT, and NG, respectively. Our present findings on HSV, CT, and NG infection are consistent with previous surveys on the prevalence of STDs. More recently, similar survey found HSV-2 DNA in 7% of 509 women in the USA [11]. Earlier study in Turkey showed that 12.7% of the low risk women were infected with CT [16]. Gaydos et al. reported that 3.8% of their female studied populations were infected with NG [17].

Previous literatures indicated that the majority of HSV, CT, and NG infections may be asymptomatic and with a long-term duration [4, 25, 26]. Similarly, in the present study, none of the women was aware of her infection before undergoing screening; however, some clinical symptoms were visible. Prevalence of STDs was consistently more in high risk populations compared with those considered at a lower risk. Commonly cited risk factors associated with STDs include unmarried status and multiple sexual partners [12]. In Amsterdam, The Netherlands, the highest prevalence of HSV-1 or HSV-2 was noticed in the youngest age groups while teenagers and adults in the twenties had a prevalence of 5.26% and 4.31%, respectively [8]. In Peru, the prevalence of infection in women with CT infection was 6·5%, and with NG infection was 0·1% [19]. In South Korea, the overall detection rate for CT was 2.4% and for HSV type II was 0.8% [20]. In Tunisian female sex workers, CT, NG, and HSV-2 PCR were positive in 72.9%, 11.2%, and 1.1% of women, respectively [21].

Although previous studies have shown that young age is associated with positive results for CT, there are some surveys that reported no correlation between age and the prevalence of disease [16]. Similarly, our findings showed that there was no significant difference in prevalence of CT between different age groups.

Various studies showed that HSV prevalence consistently increased with age in most geographic areas [27]. As expected, the results of the present study indicated that the prevalence of HSV was significantly higher in group III when compared with group I. A similar study in Bangladesh revealed that the prevalence of HSV-2 antibodies was low among married women younger than 20 years old [22]. Consequently, this prevalence increased with age which might be related to the duration of sexually active years in women.

In contrast, our findings demonstrated that the prevalence of NG decreased with age and there was a significant difference in prevalence of the infection between groups I and III and the infection was more prevalent in young women. This result is inconsistent with another study [23]. In Nigeria, NG infection was more common in women at age of 25 [23].

A study in China among female sex workers demonstrated that 8% of the studied populations were infected with NG [18] which was near to the rate of infection in young women. Although all of the women in the present study were married and they did not have sexual relationships outside the family, the prevalence of STDs in these patients was high. However, the sexual relationship of the partner needs to be evaluated. Earlier survey in France indicated that among 111 asymptomatic male partners of infertile couples, CT was detected by the PCR COBAS AMPLICOR and serology tests in 6.3% and 4.5% of patients, respectively [28]. Therefore, it seems that monitoring of sexual partners is essential for prevention of STDs.

Fungal-bacterial cervical infection was found in 45.3% of the studied population, but there were no significant differences in prevalence of HSV, CT, and NG infections between fungal-bacterial cervical infection group and non-fungal-bacterial one. It was shown that mixed infections with bacterial or fungal vaginosis and STDs may happen such as HSV, CT, NG, and* T. vaginalis* [24, 29, 30]. Additional investigations are needed to evaluate comprehensively the role of bacterial and fungal cervical infection in the pathogenesis of HSV, CT, NG, and other STDs. Monitoring the prevalence and incidence of STDs among the population and especially among youngsters would help the governmental authorities to perform and evaluate the preventive strategies and efforts. Because of considerable and long-lasting impact of STDs on quality of life and the economic loss of STDs due to high medical costs, there is a need for an accurate evaluation of STDs all over the world. Jerman et al. reported that 1.1 million new cases of STDs occurred among young patients in California in 2005, with a direct medical cost of 1.1 billion US$ [31]. Moreover, symptomatic STDs are merely the tip of the iceberg and most of these diseases are asymptomatic. So screening programs on STDs are important in all countries to prevent the transmission of the disease and help the scientists to plan for new treatment protocols. Because of public health importance of STDs, preventing measures and education of women seem necessary.

## Figures and Tables

**Table 1:** Different nationalities of studied population enrolled for detection of *Herpes simplex* virus, *Chlamydia trachomatis*, and *Neisseria gonorrhoeae* infection among married Women, Dubai, UAE.

Country	Number	Percent
Iran	82	40.8
Indonesia	2	1.0
Philippines	15	7.5
UAE	25	12.4
Egypt	8	4.0
Oman	7	3.5
Canada	1	0.5
France	2	1.0
India	9	4.5
Jordan	3	1.5
Pakistan	5	2.5
Syria	3	1.5
Afghanistan	6	3.0
China	2	1.0
Ethiopia	2	1.0
Sri Lanka	1	0.5
Lebanon	1	0.5
Thailand	1	0.5
Nigeria	3	1.5
Somalia	3	1.5
Iraq	5	2.5
Russia	2	1.0
Togo	1	0.5
Congo	1	0.5
Uzbekistan	1	0.5
Sweden	1	0.5
Australia	2	1.0
Algeria	2	1.0
Bangladesh	1	0.5
USA	1	0.5
Morocco	1	0.5
Sudan	2	1.0

Total	201	100

**Table 2:** Prevalence of *Herpes simplex* virus (HSV), *Chlamydia trachomatis* (CT), and *Neisseria gonorrhoeae* (NG) in different age groups among married Women, Dubai, UAE.

Age groups (years)	Number of positive cases (%)		
	NG	CT	HSV
15–30	7/99 (7.1)	7/99 (7.1)	1/99 (1)
31–40	4/54 (7.4)	6/54 (11.1)	3/54 (5.5)
≥41	0/48 (0)	8/48 (16.7)	9/48 (18.8)

Total	11/201 (5.5)	21/201 (10.5)	13/201 (6.5)

**Table 3:** Comparison of our data in Dubai, UAE, for prevalence of *Herpes simplex* virus (HSV), *Chlamydia trachomatis* (CT), and *Neisseria gonorrhoeae* (NG) with different studies.

Study	Number of positive cases (%)		
	NG (%)	CT (%)	HSV (%)
Our study, Dubai, UAE	5.5	10.5	6.5
Simms et al. (2003), UK [3]	—	27	—
Vahidnia et al. (2013), Amsterdam, The Netherlands [8]	—	—	5.26 (HSV-1) 4.31 (HSV-2)
Xu et al. (2006), USA [9]	—	—	57.7 (HSV-1) 17.0 (HSV-2)
Wang et al. (2012), China, Hekou, Yunnan Province [10]	—	—	58.3 (HSV-2)
Aumakhan et al. (2010), USA [11]	—	—	7 (HSV-2)
Miller et al. (2000), USA [12]	—	7.8	—
Geraats-Peters et al. (2005), The Netherlands [14]	30	—	—
Tosun et al. (2008), Turkey [16]	—	12.7	—
Gaydos et al. (2010), USA [17]	3.8	8.9	—
Wang et al. (2008), China, Yunnan Province [18]	8	26	68 (HSV-2)
Cárcamo et al. (2012), Peru [19]	0.1	6.5	13.6 (HSV-2)
Choi et al. (2012), South Korea [20]	—	2.4	0.8 (HSV-2)
Znazen et al. (2010), Tunisia [21]	11.2	72.9	1.1 (HSV-2)
Bogaerts et al. (2001), Dhaka, Bangladesh [22]	—	—	12 (HSV-2)
Franceschi et al. (2007), Spain and Nigeria [23]	0.2–6	0.2–5.6	—
Madhivanan et al. (2008), Mysore, India [24]	—	8.2	11.2 (HSV-2)
